# Differential composition of lymphocyte subpopulations and activation between the hypertensive Bph/2 and normotensive Bpn/3 mouse strains

**DOI:** 10.3389/fimmu.2025.1598843

**Published:** 2026-01-23

**Authors:** Devon Dattmore, Paiton O. McDonald, Afrin Chowdhury, Saamera Awali, Allison P. Boss, Yining Jin, Lisa Sather, D. Adam Lauver, Cheryl E. Rockwell

**Affiliations:** 1 Applied Immunology Center for Education and Research, Michigan State University, East Lansing, MI, United States; 2 Department of Pharmacology and Toxicology, Michigan State University, East Lansing, MI, United States; 3 Department of Animal Science, Michigan State University, East Lansing, MI, United States; 4 Integrated Pharmacological Sciences Training Program, Michigan State University, East Lansing, MI, United States; 5 Department of Food Science and Human Nutrition, Michigan State University, East Lansing, MI, United States

**Keywords:** B cells, Bph/2 mice, hypertension, immune cells, lymph nodes, spleen, T cell activation, T cells

## Abstract

**Introduction:**

Numerous studies point to a role for the immune system in various animal models of hypertension. However, little is known about the immune system of Bph/2 mice, a spontaneously hypertensive strain.

**Method:**

To address this, we conducted a comprehensive comparison of immune cell composition and response to polyclonal T cell activation in hypertensive Bph/2 mice and normotensive Bpn/3 control mice. We quantified immune cell populations by flow cytometry from spleen and inguinal, brachial and mesenteric lymph nodes.

**Results:**

While composition of myeloid immune cell types was largely comparable between strains, we observed differences in B and T cell subpopulations. Specifically, we found an increased percentage of IgM+ IgD^Lo^ and IgM+ IgD- B cells in Bph/2 mice, suggesting greater baseline B cell activation or differences in B cell lineage. In addition, we observed a decreased percentage of CD4 effector memory T cells and CD8 central memory T cells. The diminished proportion of memory T cells in Bph/2 mice correlated with decreased proliferation and cytokine response of splenic T cells to polyclonal T cell activation. In splenic T cells from Bph/2 mice 24 h after activation, we observed a pronounced decrease in the majority of T cell cytokines. At 120 h after activation, the Th1 and Th17 cytokine responses of splenic T cells from Bph/2 mice were decreased, but other T cell cytokines were largely comparable between genotypes.

**Conclusion/Discussion:**

Overall, the data suggest a decreased percentage of memory T cells in Bph/2 mice that correlates with markedly diminished proliferation and cytokine response to polyclonal activation.

## Introduction

The Bph/2 mouse strain was originally generated by Dr. Gunther Schlager with the purpose of creating a genetic model of hypertension in mice. To achieve this, Dr. Schlager crossed eight different inbred strains of mice, including LP/J, SJL/J, BALB/cJ, C57BL/6J, 129/J, CBA/J, RF/J and BDP/J (1, 2). After creating the 8-way cross, he bred the resulting progeny together for three generations before stratifying them based on blood pressure. Using this approach, Dr. Schlager created numerous mouse strains—many of which continue to be used. These include Bpl/1 mice (a low blood pressure strain), Bph/2 (a high blood pressure strain) and Bpn/3 (a normotensive control strain). The difference in blood pressure among these strains occurs early in life and is substantial. The elevated blood pressure in Bph/2 mice was found to occur in mice as young as 6 weeks of age (1). The systolic blood pressures of Bph/2 mice is more than 30 mm Hg greater than those of Bpn/3 mice (1). The difference increased to approximately 50 mm Hg when comparing Bph/2 and Bpl/1 mice. Schlager’s group also found a slightly elevated heart rate in Bph/2 mice as compared to Bpn/3 mice (1).

The development of hypertension is notably complex with roles for the sympathetic nervous system, vascular contraction/stiffness and kidney function, among other factors. In addition to these factors, the immune system is emerging as another possible element that may contribute to blood pressure regulation. Research from the 1960’s demonstrated that immunosuppressive drugs were protective in certain animal models of hypertension and that immune cells from hypertensive animals can transfer hypertension into non-hypertensive animals (3, 4). More recent studies have shown that angiotensin II-driven hypertension is diminished in Rag 1 knockout mice, a strain that lacks B cells and T cells (5). Likewise, the DOCA salt model of hypertension is also less pronounced in Rag1-/- mice. There have been analogous studies performed in rat models demonstrating that high salt-driven hypertension in Dahl S rats is decreased in the absence of Rag1 or CD247, a gene necessary for a functional T cell receptor (6, 7).

While these data point to a role for the immune system in at least some models of hypertension, the immune system in Bph/2 mice has not been well characterized. Thus, the purpose of the present study was to address this. Toward this end, we collected leukocytes from several different lymphoid compartments, including spleen and inguinal, brachial and mesenteric lymph nodes. We compared immune cell composition between the Bph/2 (hypertensive) and Bpn/3 (normotensive) strains. We also investigated potential strain differences in immune cell activation and cytokine production in splenocytes.

## Methods

### Materials

The purified anti-CD3 and anti-CD28 antibodies were purchased from BD Biosciences (Franklin Lakes, NJ). The cross-linker antibody was obtained from Jackson Immunoresearch (West Grove, PA). The conjugated antibodies for flow cytometry were purchased from either Invitrogen (Waltham, MA) or Biolegend (San Diego, CA), as indicated in the table below. All other materials were obtained from Millipore Sigma-Adrich (St. Louis, MO) unless otherwise indicated.

### Animals

Male Bph/2 and Bpn/3 mice were purchased from Jackson Laboratories (Bar Harbor, ME). The animals were housed in specific pathogen free conditions for the duration of the study and allowed to acclimate for a week prior to the start of experiments. The mice were provided food and water ad libitum and maintained on a 12 h light-dark cycle. Tissues were collected from the mice at 12 weeks of age. This study protocol was approved by the Institutional Animal Care and Use Committee (IACUC) at Michigan State University and conducted in accordance with the NIH Guide for the Care and Use of Animals.

### Tail-cuff plethysmography

Blood pressure was measured in conscious mice by tail-cuff plethysmography using a RTBP1001 tail-cuff blood pressure system (CODA-6, Kent Scientific, Torrington, CT). All mice were acclimatized to both handling and the blood pressure measurement system ahead of the experimental measurements. Of the 25 cycles measured, the first 15 were used as acclimation, and the remaining 10 were averaged to provide the final data point for each animal.

### Tissue collection

Spleen and inguinal, brachial and mesenteric lymph node tissues were aseptically collected from 12-week-old Bpn/3 or Bph/2 mice and processed into single-cell suspensions. The cells from inguinal, brachial and mesenteric lymph nodes were immediately analyzed via flow cytometry. Splenocytes were divided into two fractions: one that was immediately analyzed via flow cytometry and another that was cultured for immune cell activation and subsequent analysis.

### Activation of splenocytes

Splenocytes were activated with anti-CD3/anti-CD28/crosslinker (1.5 μg/mL anti-CD3, 1.5 μg/mL anti-CD28, 1.5 μg/mL crosslinker) and cultured at 37 °C, 5% CO_2_ in RPMI 1640 containing 100 U penicillin/mL, 100 U streptomycin/mL, 25 mM HEPES, 10 mM nonessential amino acids, 1 mM sodium pyruvate, and 10% fetal bovine serum (Biowest LLC, Kansas City, MO). Cells were cultured in 96-well plates and collected at 24 h and 120 h post-activation for flow cytometry. Prior to flow cytometry, cell supernatants were collected and stored at -80°C for subsequent cytokine analysis.

### Flow cytometry

Cells from spleen and inguinal, brachial and mesenteric lymph nodes were labeled using three different anti-body staining panels (a broad immune panel, a T cell-specific panel, and a B cell-specific panel). Total counts averaged approximately 300,000 – 400,000 per panel for the Bpn/3 mice and 100,000 per panel for the Bph/2 mice (who have smaller lymphoid organs). Splenic T cells activated with anti-CD3/anti-CD28 were analyzed using the T cell-specific panel (24-hours post activation). Cells were stained with Zombie Aqua Fixable Dye and labeled as described previously (8). The antibodies used are listed in **Tables 1**–**3**. Fluorescence was detected and quantified by an Attune NxT flow cytometer (Thermo Fisher Science Inc., Waltham, MA).

**Table 1:** Broad immune panel.

Antigen	Fluorochrome	Dilution	Manufacturer
CD3	AF488	1:100	Biolegend
CD4	APC/Cy7	1:100	Biolegend
CD8	PE/Cy5.5	1:100	Invitrogen
CD11b	BV605	1:100	Biolegend
CD11c	AF647	1:100	Biolegend
CD19	PE/Dazzle594	1:100	Biolegend
CD45	PerCP/Cy5.5	1:100	Biolegend
F4/80	AF700	1:100	Biolegend
Gr-1	PacBlue	1:100	Biolegend
I-Ab	PE/Cy7	1:100	Biolegend
NK1.1	BV711	1:100	Biolegend
SiglecF	PE	1:100	Biolegend

**Table 2:** B cell panel.

Antigen	Fluorochrome	Dilution	Manufacturer
CD19	PE/Dazzle594	1:100	Biolegend
CD23	PacBlue	1:100	Biolegend
CD69	PE/Cy7	1:100	Biolegend
CD86	APC/Cy7	1:100	Biolegend
IgD	BV605	1:100	Biolegend
IgM	AF488	1:100	Biolegend
CD138	PE	1:100	Biolegend

**Table 3:** T cell panel.

Antigen	Fluorochrome	Dilution	Manufacturer
CD3	AF488	1:100	Biolegend
CD4	APC/Cy7	1:100	Biolegend
CD8	PE/Cy5.5	1:100	Invitrogen
CD25	BV711	1:100	Biolegend
CD44	PE/Dazzle594	1:100	Biolegend
CD62L	PacBlue	1:100	Biolegend
CD69	AF700	1:100	Biolegend
CD134	BV605	1:100	Biolegend
CD137	PE/Cy7	1:100	Biolegend
CD154	APC	1:100	Biolegend

### Cell proliferation

Splenocytes (25,000 cells/well) in a 96-well plate were labeled with IncuCyte Nuclight Rapid Red Cell Labeling kit following the manufacturer’s protocol (Sartorius, Gottingen, Germany). Fluorescence was quantified every 8 h using the Incucyte S3 Live-Cell Analysis System (Sartorius, Gottingen, Germany).

### Cytokine analysis

Supernatants were shipped to Eve Technologies (Calgary, AB) for cytokine analysis. Each sample was analyzed by multiplex bead array using the MD32 and MD12-TH17 kits.

### Statistical analysis

The mean ± SEM was determined for each treatment group. For analyses with two experimental groups, a Student’s t test was used (Graphpad Prism 10). For analyses with more than two experimental groups and normally distributed data, a one-way or a two-way ANOVA was used (Graphpad Prism 10). For analyses with that were not normally distributed with multiple groups, the data were first log transformed prior to analysis by one-way or two-way ANOVA and a Dunnet’s *post hoc* test using Sigmaplot v12 (Systat Software, San Jose CA). Five animals were included for each treatment group, which was determined by power analysis balanced by an institutional requirement to use the fewest animals possible. The power analysis was conducted on parameters that are known to be different between the two strains: mean arterial pressure (power: 0.96), systolic blood pressure (power: 0.82) and diastolic blood pressure (power: 0.9).

## Results

### Lower body weight, but increased blood pressure, in Bph/2 mice

**Figure 1:**
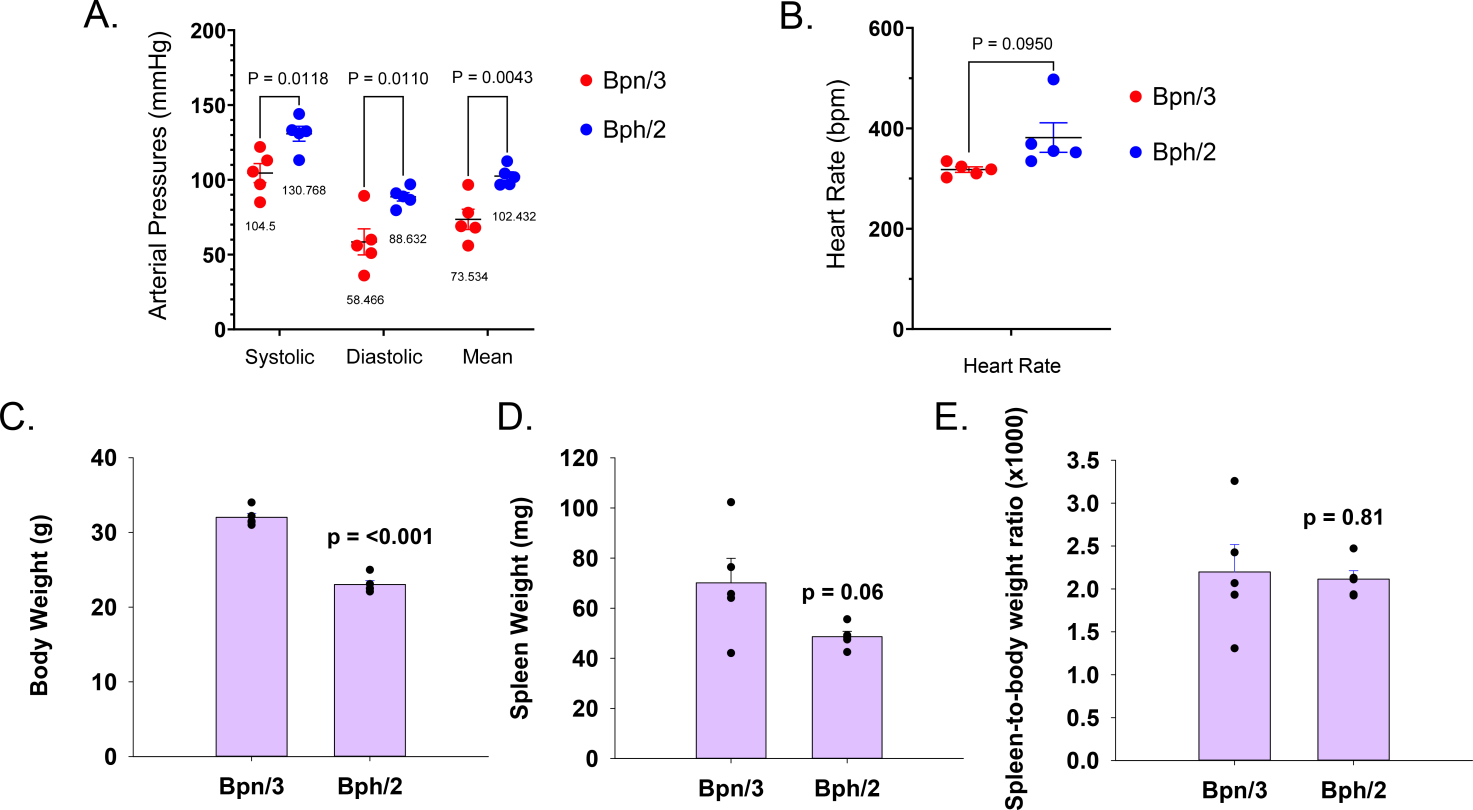
Bph/2 mice have elevated arterial blood pressure and lower body weights. Blood pressure was determined by tail cuff plethysmography. Shown in the graphs are the **(A)** systolic, diastolic, and mean arterial pressures, **(B)** heart rates and **(C)** body weights, **(D)** spleen weights, and **(E)** spleen-to-body weight ratio for Bpn/3 and Bph/2 mice. Data are presented as scatter plots or scatter plots plus bar graphs and include the mean ± SEM (n = 5). p values were determined by unpaired Student's t test.

We quantified blood pressure by tail cuff measurement. We opted for tail cuff measurements in this study instead of telemetry due to our concern regarding the impact of telemeter implants on baseline inflammation. As expected, Bph/2 mice had significantly elevated blood pressures as compared to the normotensive Bpn/3 control strain (**Figure 1A**). In contrast, heart rates were not significantly different between the two strains at this time point (**Figure 1B**, 10–11 weeks of age). We also observed a difference in body weight in which the Bpn/3 mice had significantly greater body mass as compared to Bph/2 mice (**Figure 1C**). Likewise, we observed a proportional difference in spleen weights such that the spleen-to-body weight ratio was equal between the two strains (**Figures 1D, E**).

### With the exception of neutrophils, composition of myeloid cell populations is largely comparable between Bph/2 and Bpn/3 mice

**Figure 2:**
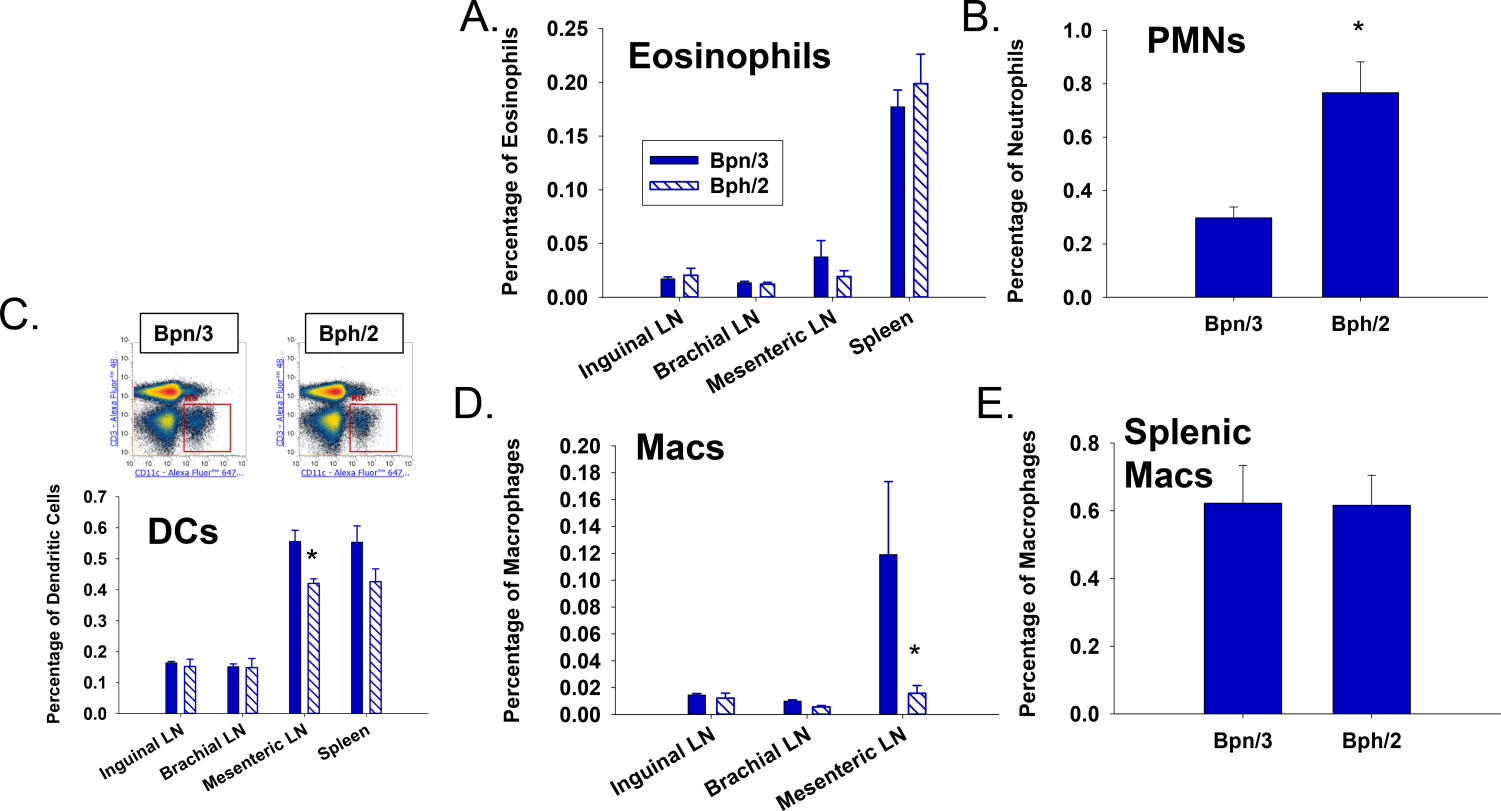
Bpn/2 mice have elevated percentages of splenic neutrophils and decreased percentages of macrophages and dendritic cells in the mesenteric lymph nodes. Splenocytes and lymph nodes (brachial, inguinal, and mesenteric) were isolated from twelve-week-old Bph/2 and Bpn/3 mice, labeled with fluorochrome-conjugated antibodies (**Table 1**) and analyzed by flow cytometry. The graphs depict **(A)** eosinophils, **(B)** neutrophils (PMNs, splenic only), **(C)** dendritic cells (y-axis: CD3-AF488; x-axis: CD11c-AF647), **(D)** macrophages, and **(E)** splenic macrophages as percentages of the live cell population. The graphs represent the mean ± SEM values for each respective group, n = 5.* p<0.05 as determined by unpaired Student’s t-test.

We assessed the composition of various immune cell populations across multiple immune compartments, including spleen as well as inguinal, brachial and mesenteric lymph nodes. Of the granulocyte populations, the percentage of eosinophils was fairly comparable between Bpn/3 and Bph/2 mice in the different compartments (**Figure 2A**). In contrast, the percentage of neutrophils in the spleens of Bph/2 mice was significantly greater than those from Bpn/3 mice (**Figure 2B**). Although we were able to quantify neutrophils in spleen, it should be noted that we were unable to quantify neutrophils in the lymph nodes due to low numbers. With respect to the antigen presenting cells, there was a modestly lower percentage of dendritic cells in mesenteric lymph nodes of Bph/2 mice as compared to Bpn/3 (**Figure 2C**). Likewise, we also observed a decreased percentage of macrophages in mesenteric lymph nodes of Bph/2 mice (**Figure 2D**). However, there were no differences in the proportion of dendritic cell or macrophage populations in spleen, inguinal or brachial lymph nodes (**Figures 2C–E**). Taken together, the data indicate that Bph/2 mice have more than double the percentage of splenic neutrophils compared to Bpn/3 mice, whereas most of the other myeloid populations were comparable between Bph/2 and Bpn/3 mice.

### Bpn/3 mice have a decreased CD4/CD8 ratio in inguinal and brachial lymph nodes

**Figure 3:**
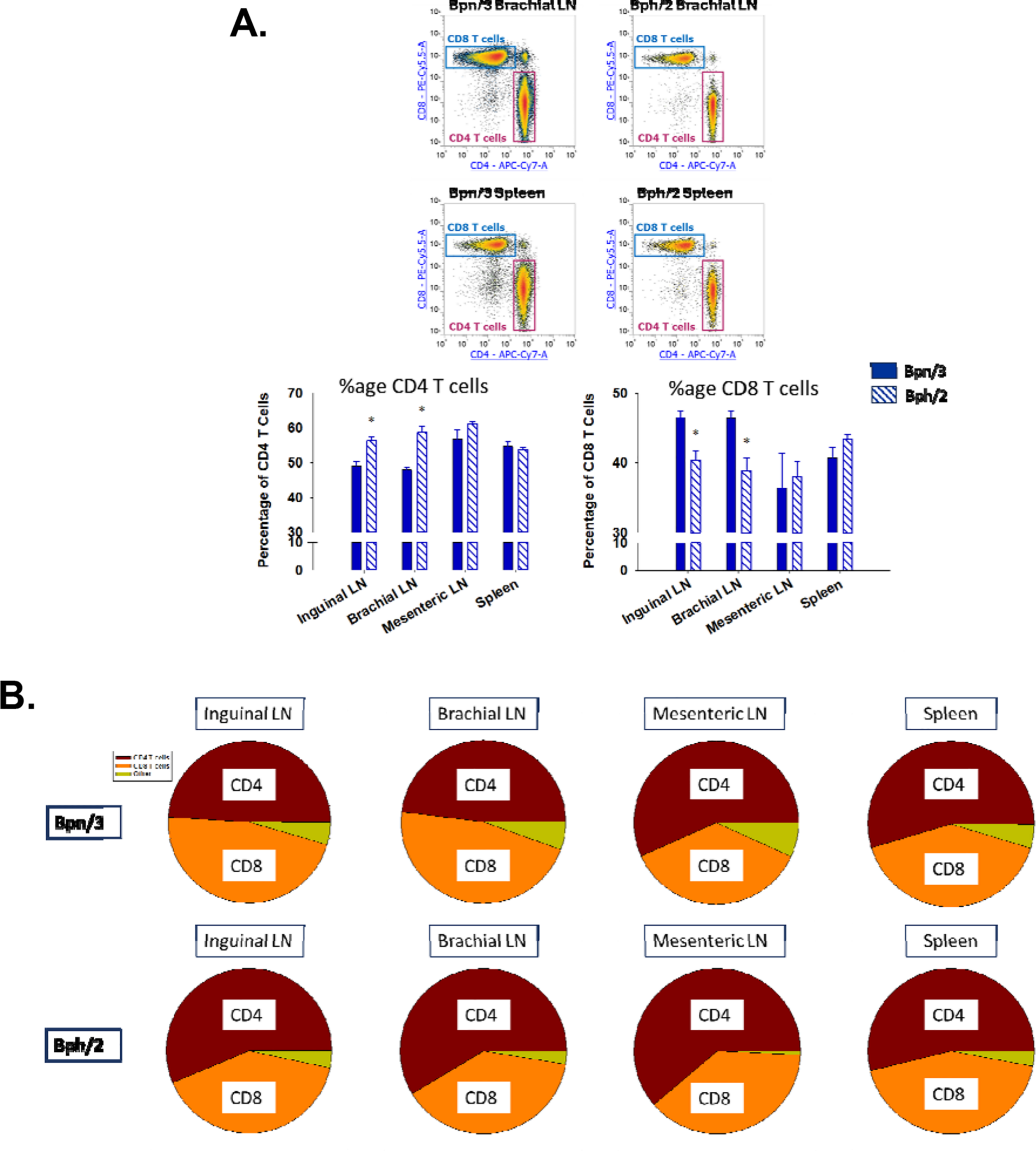
Bph/2 mice have altered percentages of CD4^+^ and CD8^+^ T cells in brachial and inguinal lymph nodes compared to Bpn/3 mice. Single-cell suspensions were extracted from spleens and lymph nodes of twelve-week-old Bpn/3 and Bph/2 mice, labeled with fluorochrome-conjugated antibodies (**Table 1**) and analyzed by flow cytometry. **(A)** CD4^+^ and CD8^+^ T cell percentages relative to CD3^+^ T cell population (y-axis: CD8-PE/Cy5.5; x-axis: CD4-APC/Cy7). Representative flow density plots are shown above, and graphs shown below illustrate the mean ± SEM values for each respective group, n = 5. **(B)** Pie graphs showing the relative proportions of CD4^+^ and CD8^+^ T cells in relation to the whole T cell population. Other refers to T cell populations that were CD4+ CD8+ double-positive or CD4- CD8- double-negative *p<0.05, as determined by unpaired Student’s t-test.

In wild-type C57Bl/6 mice (which is the most widely used mouse strain in immunology studies), the ratio of CD4 to CD8 T cells tends to remain fairly stable with a higher percentage of CD4 T cells relative to CD8 T cells. Consistent with this, we observed a greater proportion of CD4 T cells compared to CD8 T cells in every lymphoid compartment we investigated in Bph/2 mice (**Figures 3A, B**). The proportion of CD4 T cells was also greater than that of CD8 T cells in the spleens and mesenteric lymph nodes of Bpn/3 mice. In contrast, the percentages of CD4 T cells and CD8 T cells were largely equivalent in the inguinal and brachial lymph nodes of Bpn/3 mice (**Figures 3A, B**). Overall, we found a lower CD4 to CD8 T cell ratio in the inguinal and brachial lymph nodes of Bpn/3 mice, which suggests a possible difference in T cell development between the two strains.

### Splenic B cells from Bph/2 mice show lower expression of IgD than those derived from Bpn/3 mice

**Figure 4:**
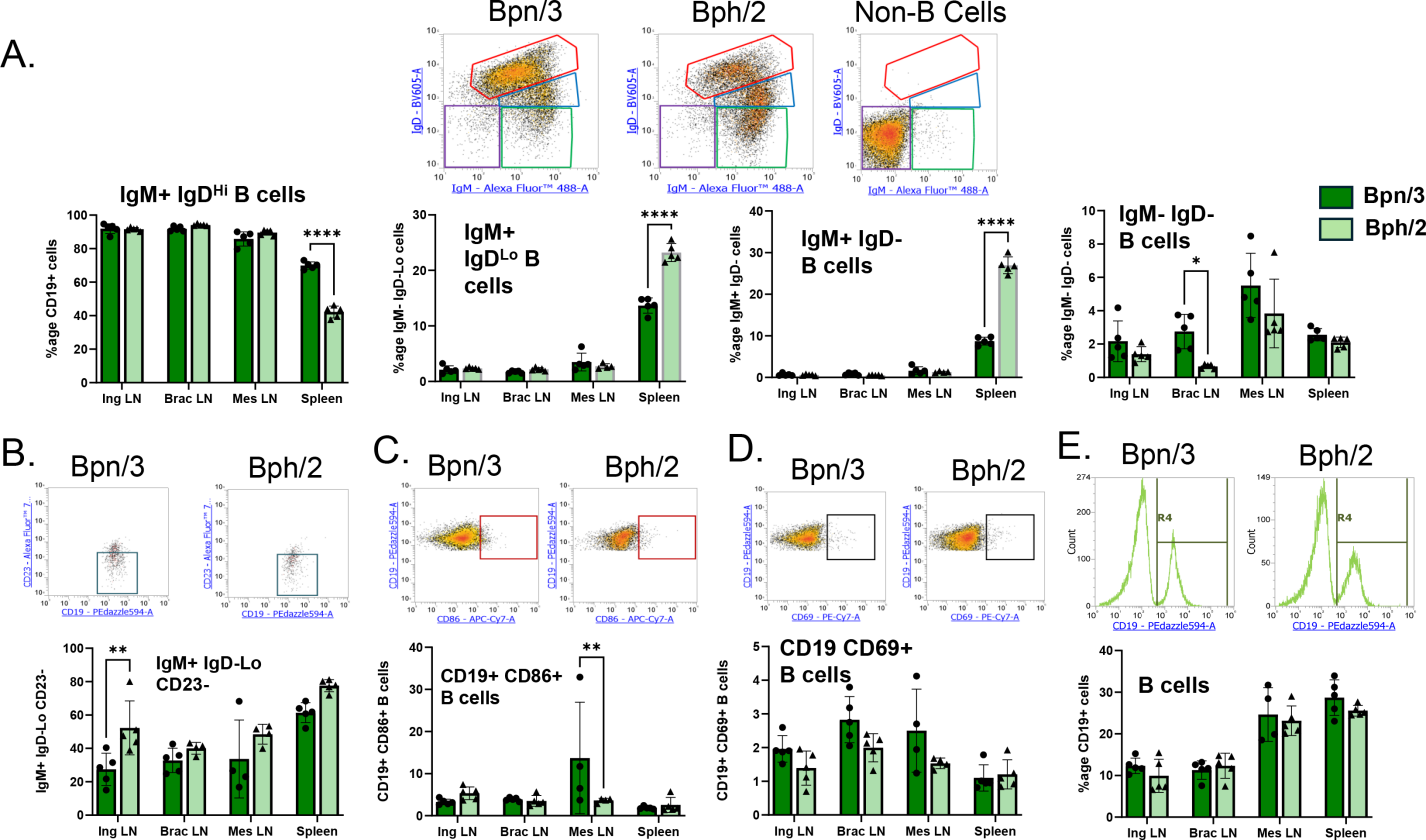
Lower percentage of IgM+ IgD-Lo and IgM+ IgD- B cells in spleens of Bph/2 mice. Single-cell suspensions were extracted from spleens and lymph nodes of twelve-week-old Bpn/3 and Bph/2 mice, labeled with fluorochrome-conjugated antibodies (**Table 2**) and analyzed by flow cytometry. The following B cell populations were quantified: **(A)** IgM^+^ IgD^Hi^ B cells (red gates), IgM^+^ IgD^Lo^ B cells (blue gates), IgM^+^ IgD- B cells (green gates), and IgM- IgD- B cells (purple gates) B cells (y-axis: IgD-BV605; x-axis: IgM-AF488), **(B)** IgM^+^ IgD^Lo^ CD23- B cells, which includes marginal zone B cells (y-axis: CD23-AF700; x-axis: CD19-PEdazzle594), and recently activated **(C)** CD86^+^ (y-axis: CD19-PEdazzle594; x-axis: CD86-APC/Cy7) and **(D)** CD69^+^ (y-axis: CD19-PEdazzle594; x-axis: CD69-PE/Cy7) B cells. **(E)** The pan B cell population (CD19^+^) was also quantified (histogram: CD19-PEdazzle594). Representative flow density plots are shown above, and graphs shown below illustrate the mean ± SEM values for each respective group, n = 5. *p<0.05 **p<0.01 and ****p<0.0001 as determined by unpaired Student’s t-test.

One of the primary functions of B cells is to express antibodies, such as IgD and IgM. Newly matured B cells express high levels of IgD and low levels of IgM. When B cells become activated, the ratio of IgM to IgD expression shifts (9, 10). Whereas fully activated B cells solely express IgM, there is a transition period shortly after the initiation of activation where B cells express high levels of IgM and low levels of IgD. We observed notable differences in splenic B cell antibody expression in Bph/2 vs. Bpn/3 mice. Specifically, we found Bph/2 spleens showed a lower percentage of IgM+ IgD^Hi^ B cells with a concurrent increased percentage of IgM+ IgD^Lo^ and IgM+ IgD- B cells. (**Figure 4A**). In contrast, the percentage of IgM- IgD- B cells was roughly comparable in spleen, but was lower in the brachial lymph nodes of Bph/2 mice. The percentage of IgM+ IgD^Lo^ CD23- B cells, which includes marginal zone B cells was equivalent between the two strains in all lymphoid compartments (**Figure 4B**). Likewise, expression of the B cell activation markers CD86 and CD69 (expressed by recently-activated B cells) was largely equivalent between strains with the exception of a low percentage of CD86+ B cells in the mesenteric lymph nodes of Bph/2 mice (**Figures 2C, D**). There were no differences in the overall pan-B cell population between the two strains (**Figure 4E**). B cells also have the ability to differentiate into plasma cells, a cell type that produces large quantities of antibody. Although we included a marker for plasma cells in our panel (CD138), the percentage of plasma cells was too low to reliably measure. Collectively, these data suggest that splenic B cells from Bph/2 mice show lower expression of IgD as compared to those from Bpn/3 mice.

### Bph/2 mice have a pronounced decrease in the effector memory CD4 T cell population as compared to Bpn/3 mice

**Figure 5:**
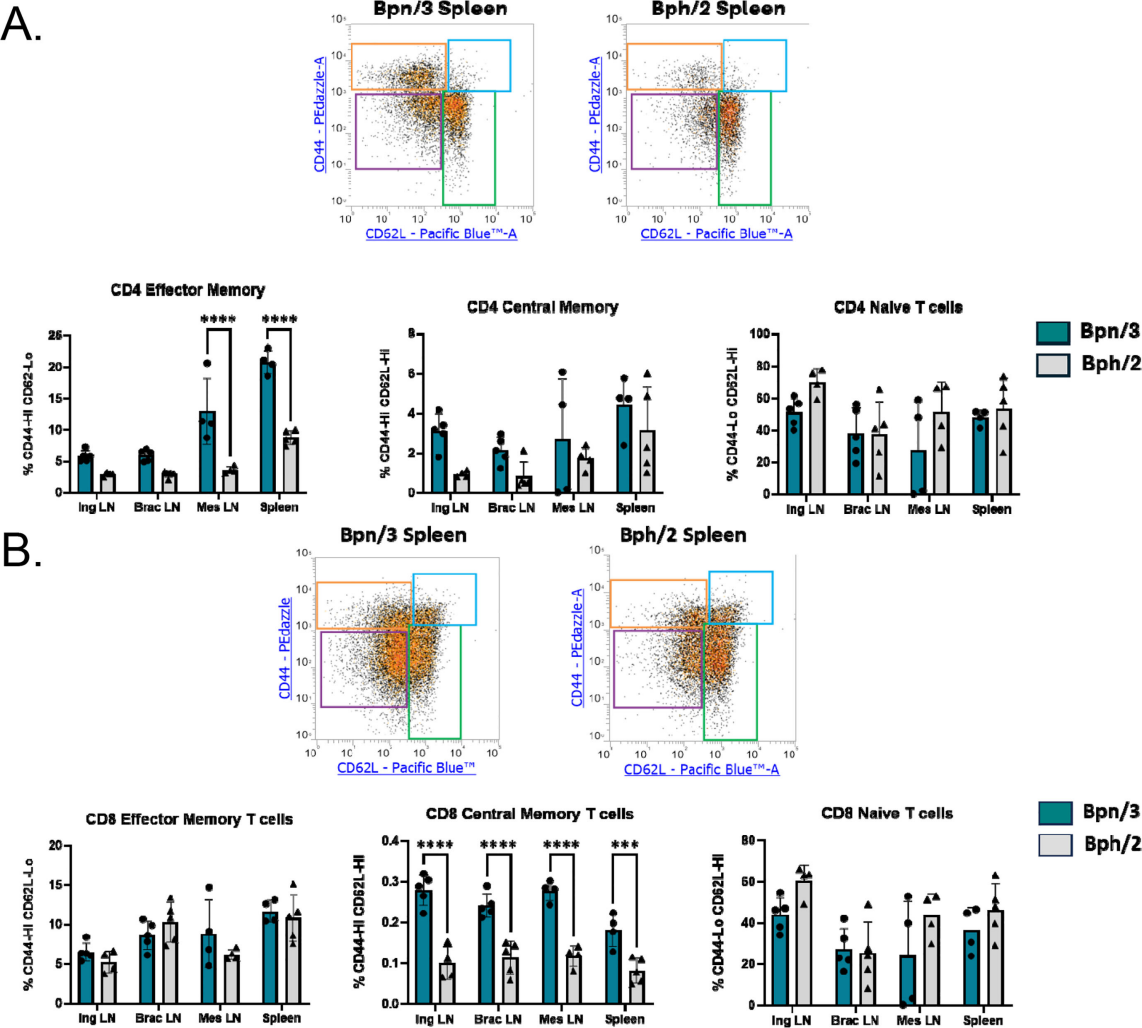
Lower percentages of CD4 effector memory T cells and CD8 central memory T cells in Bph/2 mice. Single-cell suspensions were extracted from spleens and lymph nodes of twelve-week-old Bpn/3 and Bph/2 mice, labeled with fluorochrome-conjugated antibodies (**Table 3**) and analyzed by flow cytometry (y-axis: CD44-PEdazzle; x-axis: CD62L-Pacific Blue; pre-gated on either **(A)** CD4 T cells or **(B)** CD8 T cells). Naïve (CD62L^Hi^ CD44^Lo^), effector memory (CD62L^Lo^ CD44^Hi^) and central memory (CD62L^Hi^ CD44^Hi^) T cell subsets were quantified in the A) CD4 and B) CD8 T cell populations. No differences were observed in the effector memory (CD44^Hi^ CD62L^Lo^) or naïve (CD44^Lo^ CD62L^Hi^) CD8^+^ T cells. Bars are presented as the mean ± SEM. n = 5. ***p<0.001 and ****p<0.0001 as determined by unpaired Student’s t-test.

In addition to investigating effects on CD4 and CD8 T cell ratios, we also quantified T cell memory cell populations. The two dominant memory cell populations in lymphoid organs are effector memory T cells, which have a fast, robust cytokine response to stimulation, and central memory T cells, which have an intermediate response between effector memory T cells and naïve T cells (11, 12) Naïve T cells have a slow, modest response to activation in comparison to memory cells. We found a substantially lower percentage of CD4 effector memory T cells in the Bph/2 mice as compared to Bpn/3 mice with a statistically significant difference in all lymphoid organs examined (**Figure 5A**). In contrast, we did not observe any difference in the percentages of effector memory T cells within the CD8 T cell population, but rather, we observed a significantly lower percentage of central memory CD8 T cells that was consistent across all compartments (**Figure 5B**). Taken together, the data indicate a reduction in the proportion of memory T cell populations in Bph/2 mice.

### Diminished proliferation in splenic T cells from Bph/2 mice activated with a polyclonal activator

**Figure 6:**
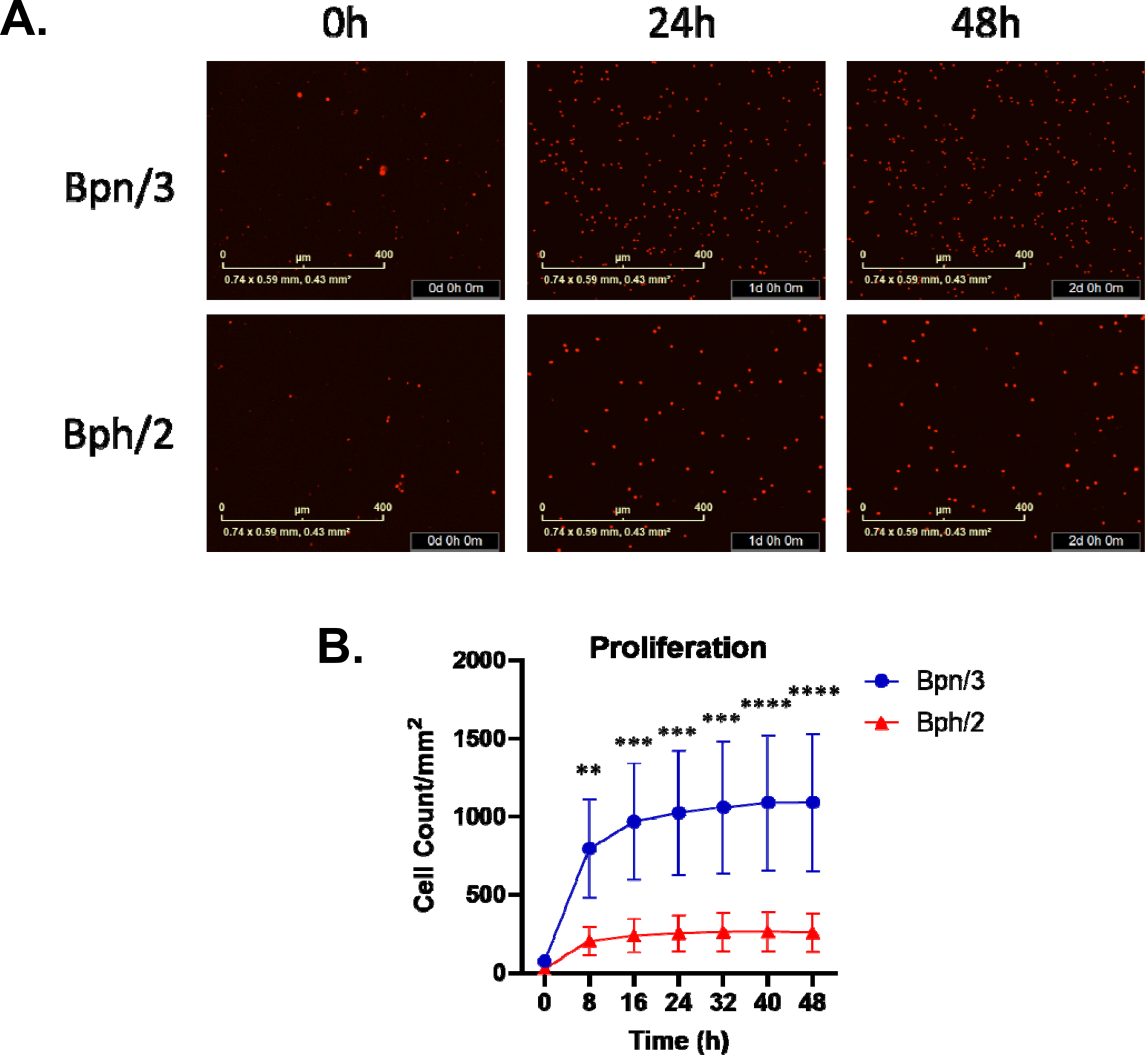
Decreased proliferation by activated splenic T cells from Bph/2 mice. Freshly isolated splenocytes were (2.5x10^4^ cells/well), stained with IncuCyte Nuclight Rapid Red reagent, and cultured for 48 (h) Fluorescence was quantified every 8 h using the Incucyte S3 Live-Cell Analysis System. **(A)** Representative images of cells from Bph/2 and Bpn/2 mice. **(B)** Line graph depicting cell counts at 0–48 h post activation. The data are presented as the mean ± SEM, n=5. **p<0.01, ***p<0.001 and ****p<0.0001 as determined by 2-way ANOVA followed by Dunnett's post hoc analysis.

In addition to assessing baseline homeostatic differences in immune cell composition between Bph/2 and Bpn/3 mice, we also compared the impact of polyclonal T cell activation (anti-CD3/anti-CD28) on splenic T cells from the two strains. For these studies, we used a freshly isolated splenocyte preparation activated with anti-CD3/anti-CD28. T cell activation results in a number of functional changes, including induction of proliferation, upregulation of certain cell surface receptors and an enormous increase in cytokine secretion. Notably, we observed markedly decreased proliferation in the cells derived from Bph/2 mice as compared to those from Bpn/3 mice (**Figures 6A, B**).

### Decreased upregulation of activation markers in CD8 T cells from Bph/2 mice

**Figure 7:**
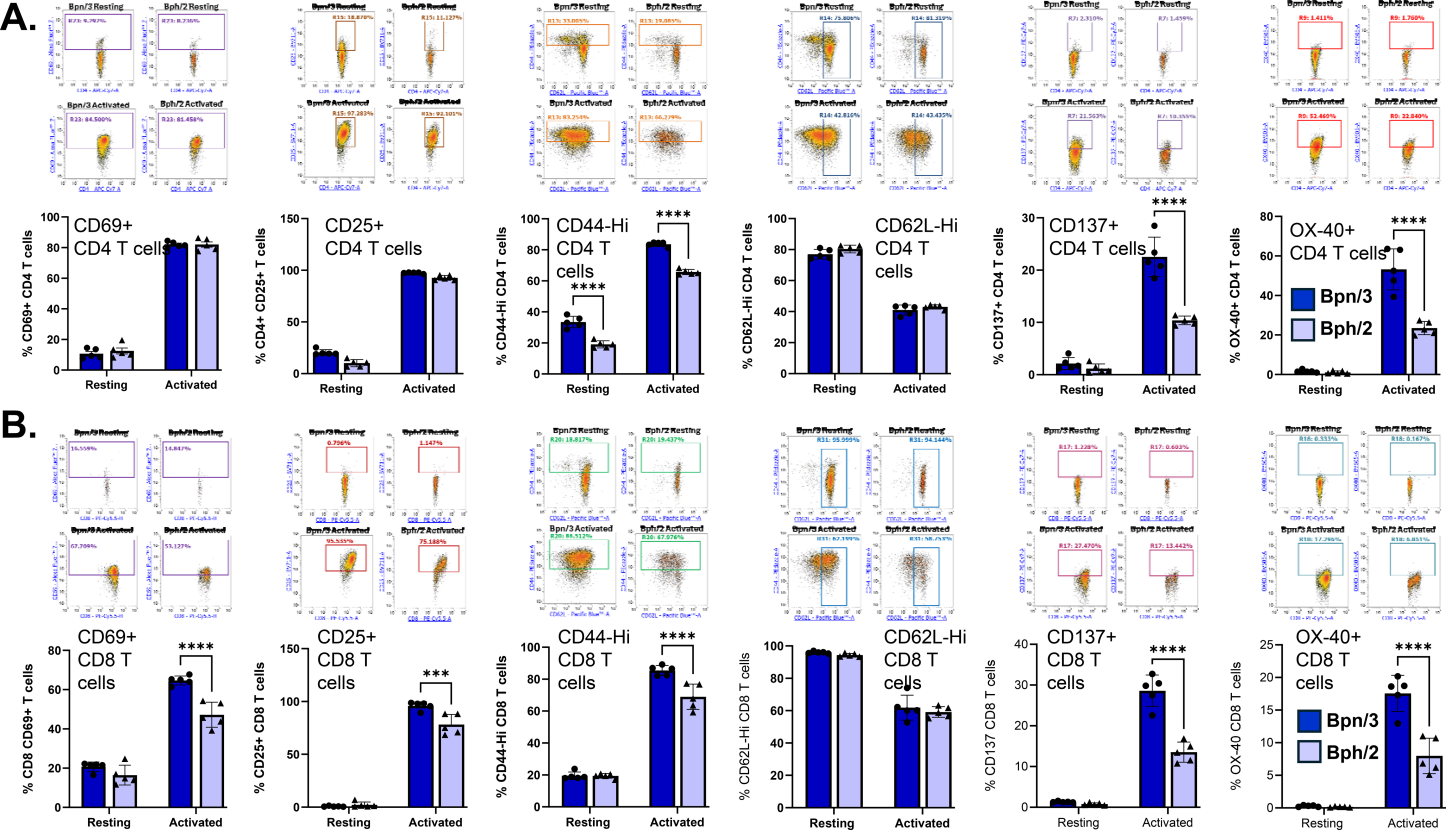
Diminished induction of T cell activation markers in CD8 T cells, and to a lesser extent, CD4 T cells from Bph/2 mice. Freshly isolated splenocytes were cultured in the presence or absence anti-CD3/anti-CD28 activating antibodies for 24h. The cells were then labeled with fluorochrome-conjugated antibodies (**Table 3**) and analyzed by flow cytometry. A) Decreased percentages of CD4^+^ T cells from BPH/2J mice were CD44^Hi^(y-axis: CD44-PEdazzle; x-axis: CD62L-Pacific Blue), CD137^+^,(y-axis: CD137-PE/Cy7; x-axis: CD4-APC/Cy7) and OX-40^+^ (y-axis: OX40-BV605; x-axis: CD4-APC/Cy7) 24-hours post-activation, whereas no differences were observed in percentages of CD25^+^(y-axis: CD25-BV711; x-axis: CD4-APC/Cy7), CD69^+^(y-axis: CD69-AF700; X-axis: CD4-APC/Cy7), and CD62L^Hi^(y-axis: CD44-PEdazzle; x-axis: CD62L-Pacific Blue). B) BPH/2J mice had decreased percentages of CD8^+^ T cells expressing the inducible cell surface markers: CD69 (y-axis: CD69-AF700; X-axis: CD8-PE/Cy5.5), CD25 (y-axis: CD25-BV711; x-axis: CD8-PE/Cy5.5), CD44 (y-axis: CD44-PEdazzle; x-axis: CD62L-Pacific Blue), CD137 (y-axis: CD137-PE/Cy7; x-axis: CD8-PE/Cy5.5) and OX-40 (y-axis: OX40-BV605; x-axis: CD8-PE/Cy5.5). CD62L (y-axis: CD44-PEdazzle; x-axis: CD62L-Pacific Blue), which is rapidly downregulated after activation was also quantified. Above each plotted graph are representative dot plots. The data are presented as the mean ± SEM, n=5. ***p<0.001 and ****p<0.0001 as determined by 2-way ANOVA followed by Dunnett's post hoc analysis.

Concurrent with an increase in proliferation, T cell activation also causes a robust upregulation of an array of cell surface receptors. For this study, we quantified expression of CD69, the function of which is not fully understood, and CD25, the high-affinity subunit of the IL-2 receptor, both of which are early T cell activation markers. We also quantified CD44, which is constitutively expressed by memory T cells, but rapidly induced in naïve T cells following activation. The inducible costimulatory receptors, OX-40 and CD137, were also measured. In contrast to all the other markers that are upregulated following activation, CD62L, an adhesion molecule that directs T cells into lymph nodes, is rapidly downregulated following activation. In CD4 T cells, there was a decreased percentage of cells expressing the late activation markers, OX-40 and CD137, in the Bph/2 group (**Figure 7A**). In contrast, there were no differences in the expression of the early activation markers CD69 and CD25 in CD4 T cells between the two strains. While we observed decreased expression of CD44 in resting CD4 T cells, we know from our previous analysis, that this is likely due to a decreased effector memory CD4 T cell population. In CD8 T cells, we observed a decreased percentage of activated T cells in Bph/2 mice, which was consistent across all the inducible markers (**Figure 7B**).

### Diminished early induction of most cytokines in activated splenocytes from Bph/2 mice

**Figure 8:**
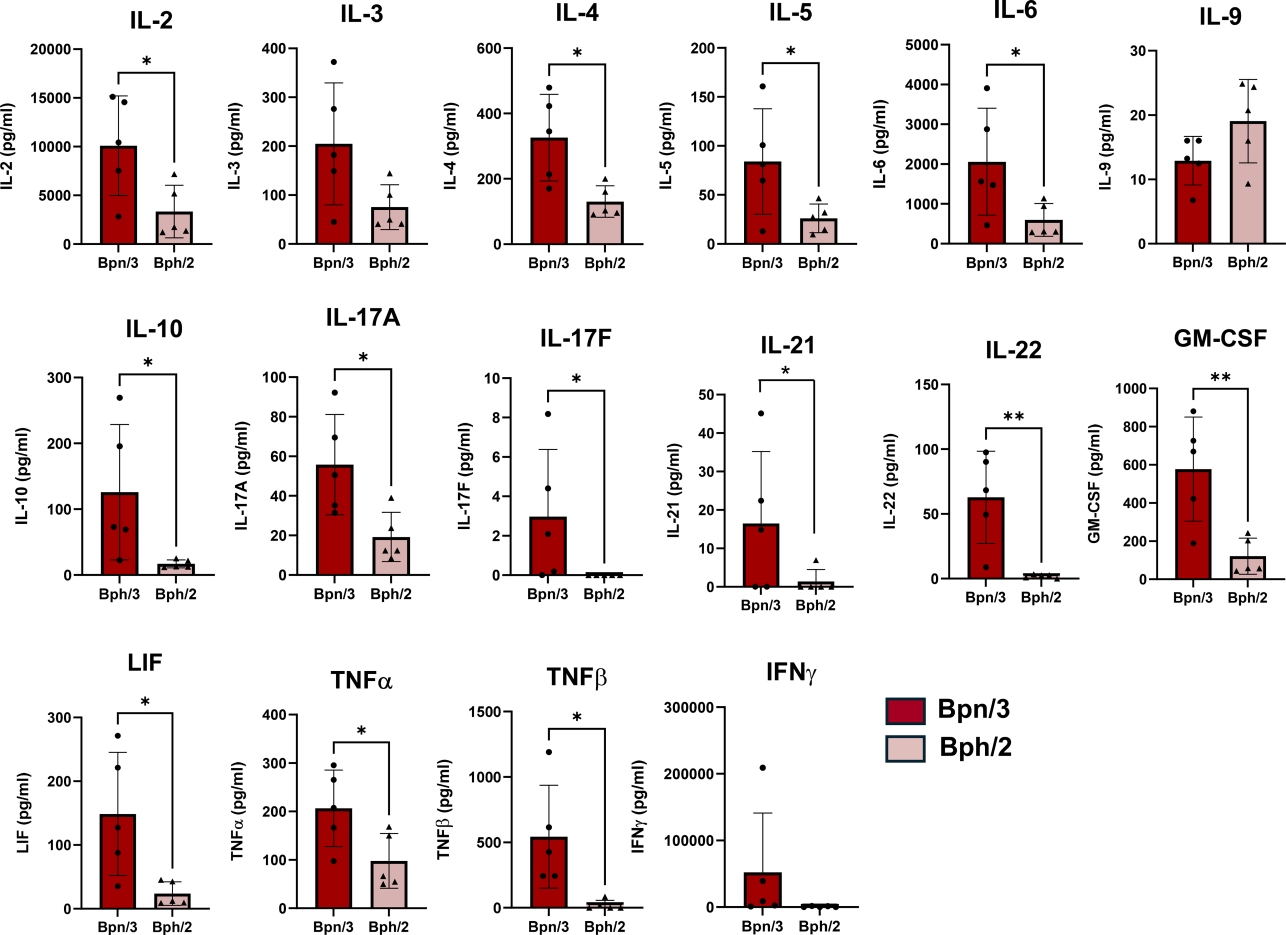
Reduced induction of T cell cytokines by splenic T cells from Bph/2 mice 24 h after activation. Freshly isolated splenocytes were activated with anti-CD3/anti-CD28 antibodies for 24 h prior to collection of cell supernatants for cytokine analysis by EVE Technologies (Calgary, Alberta, Canada). Data are presented as the mean ± SEM, n = 5. *p<0.05 and **p<0.01 as determined by two-tailed Student’s t test.

**Figure 9:**
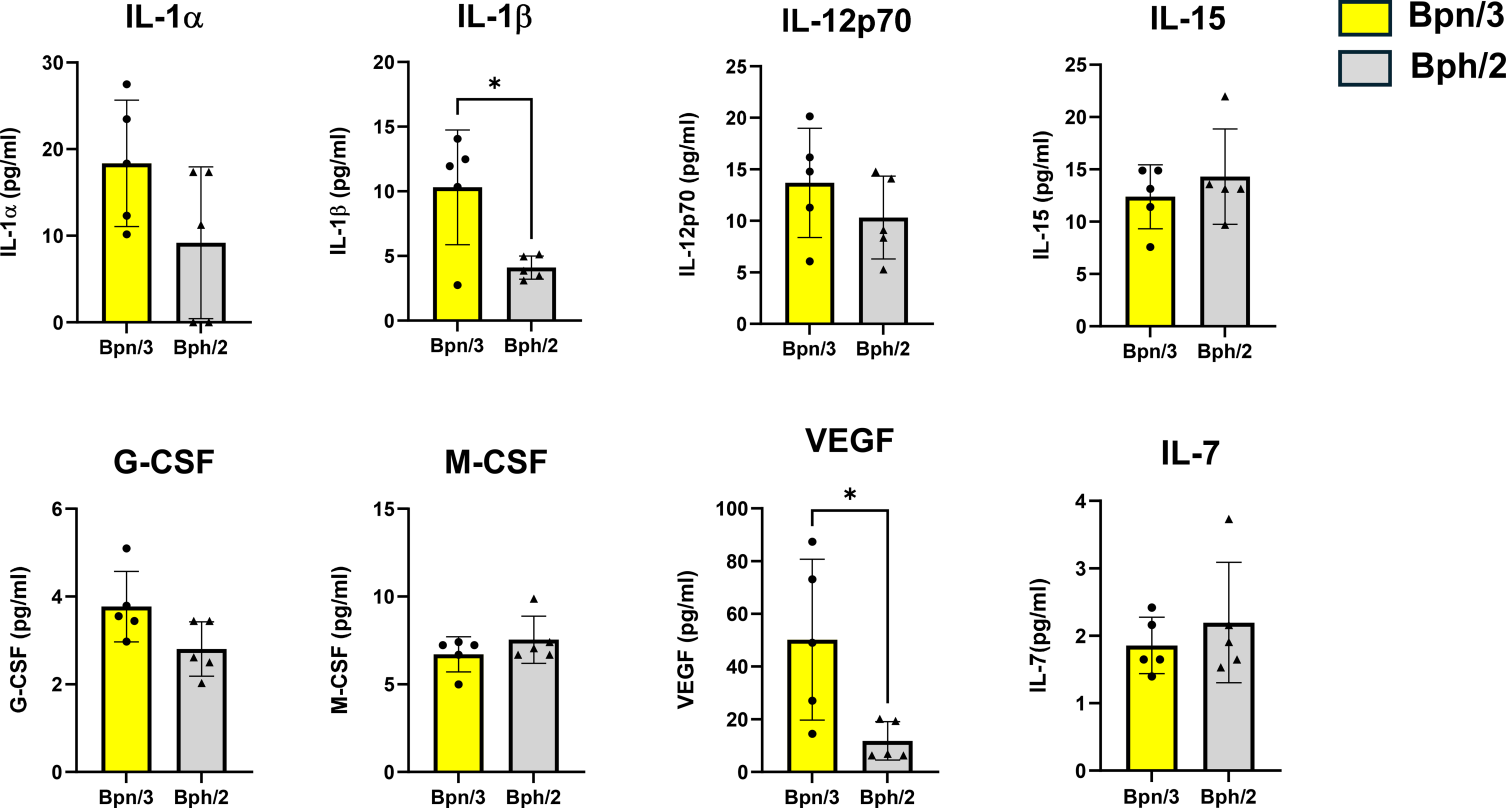
Decreased induction of IL-1β and VEGF by myeloid cells from Bph/2 mice 24 h after activation. Freshly isolated splenocytes were activated with anti-CD3/anti-CD28 antibodies for 24 h prior to collection of cell supernatants for cytokine analysis by EVE Technologies (Calgary, Alberta, Canada). Induction of cytokines was likely the result of indirect stimulation of myeloid cells by cytokines released by T cells. Data are presented as the mean ± SEM, n = 5. * p<0.05 as determined by two-tailed Student’s t test.

**Figure 10:**
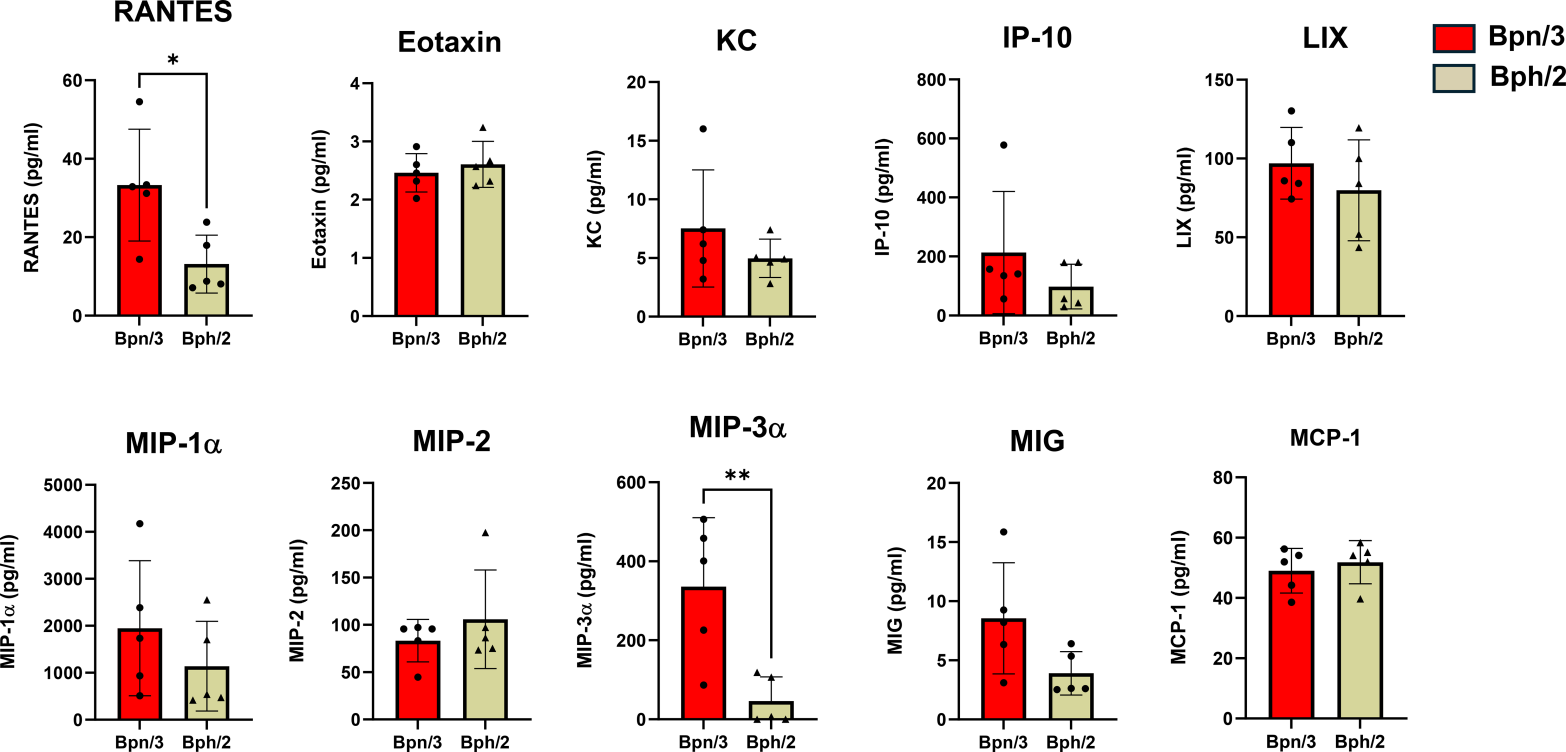
Reduced induction of the chemokines, RANTES and MIP3α, by splenic T cells from Bph/2 mice 24 h after activation. Freshly isolated splenocytes were activated with anti-CD3/anti-CD28 antibodies for 24 h prior to collection of cell supernatants for cytokine analysis by EVE Technologies (Calgary, Alberta, Canada). Data are presented as the mean ± SEM, n = 5. *p<0.05 and **p<0.01 as determined by two-tailed Student’s t test.

**Figure 11:**
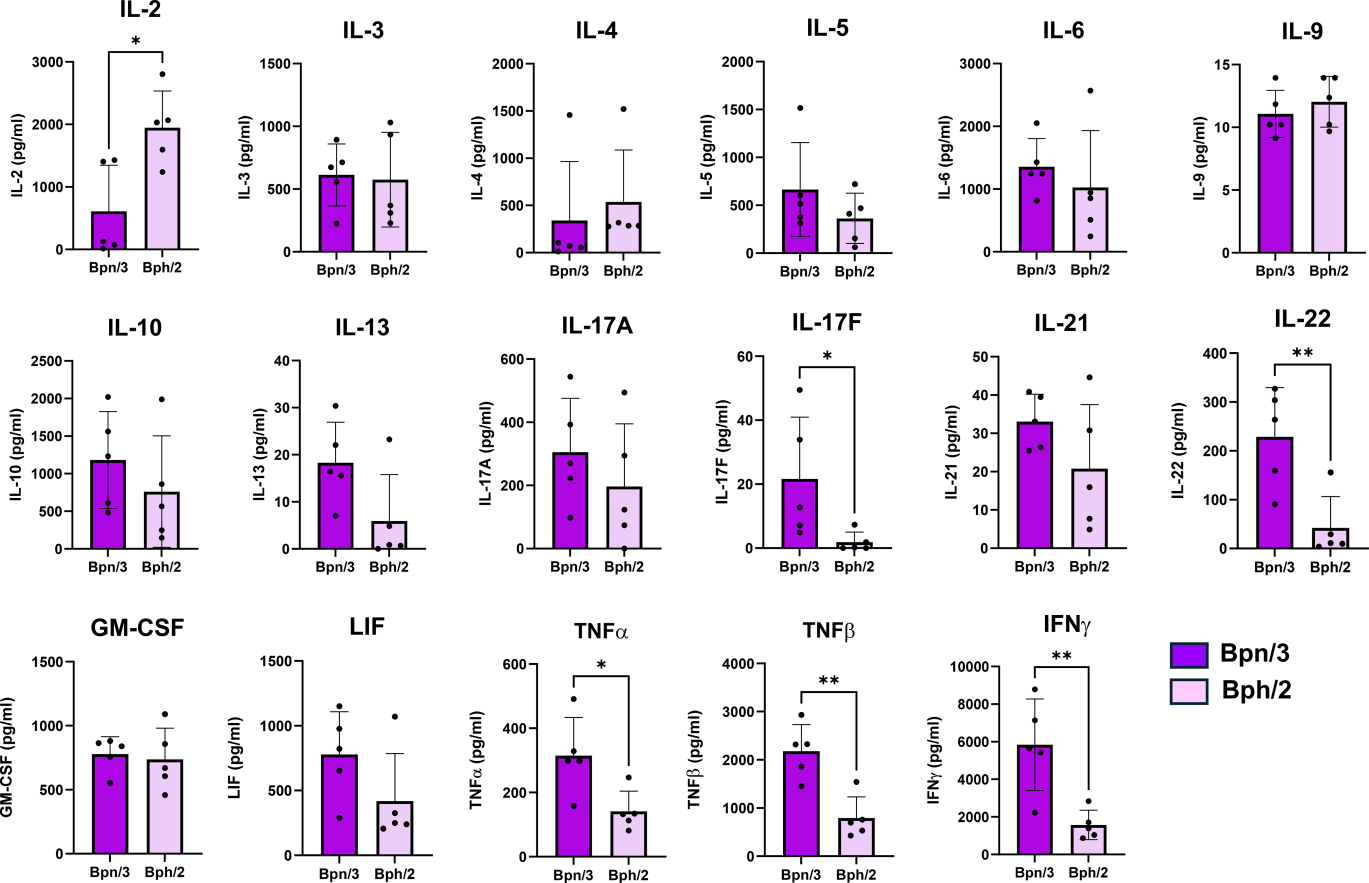
Decreased induction of T cell cytokines by splenic T cells from Bph/2 mice 120 h after activation. Freshly isolated splenocytes were activated with anti-CD3/anti-CD28 antibodies for 120 h prior to collection of cell supernatants for cytokine analysis by EVE Technologies (Calgary, Alberta, Canada). Data are presented as the mean ± SEM, n = 5. *p<0.05 and **p<0.01 as determined by two-tailed Student’s t test.

To determine differences in T cell function, we also assessed differences in cytokine production between the Bph/2 and Bpn/3 strains. Shortly after activation, T cells rapidly secrete an array of cytokines. CD4 T cells, in particular, differentiate into multiple functionally distinct subsets that are distinguished in part by the cytokines produced. We observed consistently decreased induction of T cell cytokines in the Bph/2 group 24 h after activation (**Figure 8**). At this early time point, we did not observe differences in cytokines associated with any one particular T cell lineage (Th1, Th2, Th17, etc.). While induction of most cytokines was diminished in the Bph/2 group, an exception to this was IL-9 which remained unchanged. We also noted the presence of cytokines that are not typically produced at appreciable levels by T cells. These cytokines were likely produced by macrophages and other myeloid cells that were stimulated by cytokines and costimulatory factors produced by the activated T cells (**Figure 9**). In addition, our data show upregulation of numerous chemokines (**Figure 10**). While myeloid-derived cytokines were somewhat comparable between strains, we observed significantly lower secretion of IL-1β and VEGF in activated splenocytes from Bph/2 mice (**Figure 9**). We also observed decreased levels of the MIP3α and RANTES chemokines in the Bph/2 group (**Figure 10**). Because trends in cytokine production can change over time following activation, we also looked at T cell-derived cytokines at 120 h after activation. In general, the expression of many cytokines was largely equivalent between the two strains at this time point (**Figure 11**). There were exceptions to this, however, including markedly increased IL-2, but decreased Th1 (IFNγ and TNFα) and Th17 (IL-17F, IL-22, TNFβ) cytokines in the Bph/2 group. Overall, the data point to diminished induction of T cell cytokines in activated cells from Bph/2 mice at early time points after activation with a persistent decrease in Th1 and Th17 cytokines at late time points.

## Discussion

The purpose of the present study was to compare the immune systems of the hypertensive Bph/2 mouse and normotensive Bpn/3 mice. Specifically, we compared immune cell composition in spleen and inguinal, brachial and mesenteric lymph nodes. We also assessed differences in lymphocyte response to polyclonal T cell activation. Overall, the immune cell composition was similar between the two strains with the exception of a markedly increased splenic neutrophil population in the Bph/2 mice. While overall proportion of B cells was comparable between strains, there were differences in antibody isotype expression in which there was a greater percentage of IgM+ B cells in Bph/2 mice that were either negative for, or had low expression of, IgD. We also noted a decreased percentage of effector memory cells within the CD4 T cell population and a decreased percentage of central memory cells within the CD8 T cell population of Bph/2 mice. The diminished memory T cell populations correlated with decreased proliferation and a diminished cytokine response to polyclonal T cell activation. The decreased cytokine response was most pronounced at 24 h, but we also observed significantly diminished secretion of Th1 and Th17 cytokines at 120 h. Overall, the data indicate largely comparable immune cell composition between the two strains (with some exceptions), but notable functional differences in response to polyclonal T cell activation.

Dr. Gunther Schlager found significant differences in blood pressure between different strains of mice, which suggested a role for heritable genes in hypertension (13–19). Accordingly, he originally generated the Bph/2 and the Bpn/3 mouse strains to identify the role of genetics in determining arterial blood pressure (1, 2, 20). He found that blood pressures of the Bph/2 and Bpn/3 mice diverged as early as 6 weeks of age (1). Similar to this report, we also observed significantly greater systolic, diastolic and mean arterial pressures in Bph/2 mice as compared to Bpn/3 mice. Dr. Schlager’s group also found an increase in heart rate. While our data show a trend toward an increase in heart rate in Bph/2 mice, the difference was not statistically significant. Notably, Jackson et al. has demonstrated that time of day plays a role in the magnitude of difference in blood pressure and heart rate between Bph/2 and Bpn/3 mice (21). Of note, our measurements were taken during the day and Jackson et al. showed that the greatest differences in blood pressure and heart rate were observed at night. Nonetheless, we found significantly elevated blood pressure in Bph/2 mice, which is consistent with previous studies and use of Bph/2 as a hypertensive model.

The most pronounced difference in immune composition between the two mouse strains is in the percentage of splenic neutrophils, which was greater in Bph/2 mice. The biological significance of this is not clear, since neutrophils account for a fairly small fraction of immune cells in spleen. However, the increased percentage of neutrophils in spleen may be proportional to differences in the blood neutrophil population since one of the functions of the spleen is to filter blood. Indeed, an increased proportion of blood neutrophils has been reported in Bph/2 mice as compared to Bpn/3 mice (22). It is also notable that these are data are consistent with a previous study that showed increased splenic neutrophils in diabetic and sham control Bph/2 mice as compared to Bpn/3 mice (23).

Although we did not observe differences in the percentage of B cells in any compartment within the Bpn/3 and Bph/2 strains, we did observe differences in the expression of antibodies in spleen, but not lymph nodes. Antibody expression in B cells is dynamic and reflects multiple factors in B cells, such as development, lineage, activation and others. Whereas newly mature B cells in spleen and lymph nodes express high levels of IgD, IgD expression is downregulated following B cell activation while IgM expression is maintained (9, 10). Some activated B cells may undergo class switching where both IgD and IgM expression are turned off and other antibody classes are expressed (IgG, IgA, etc. (24)). In addition to antibody production and other factors, B cells are also characterized by their anatomical location within lymphoid organs where the dominant population are the follicular B cells, which upon activation produce antibody in the follicles, and marginal zone B cells, which are innate-like B cells that provide quick responses to pathogens (25). Our data showed a decrease in the percentage of IgM+ IgD^Hi^ B cells with a concurrent increase in percentage of IgM+IgD^Lo^ and IgM+ IgD- B cell populations. Thus, these data could suggest a diminished proportion of naïve, unactivated B cells in Bph/2 mice with a concurrent increased percentage of antigen-experienced B cells (IgM+ IgD-). The significance of the differences in the IgM+ IgD^Lo^ B cell population is not clear since there are several functionally-distinct subpopulations within that group, including T1 transitional cells (transitioning to naïve, mature phenotype), regulatory B cells and marginal zone B cells (26–28). Of interest, we also observed an increase in the percentage of IgM+ IgD-Lo CD23- B cells (which includes the marginal zone B cell population) in the inguinal lymph nodes of Bph/2 mice (29). In addition, we observed a decrease in IgM- IgD- B cells in the brachial lymph nodes of Bph/2 mice, which suggests potential class switching to IgG and/or other antibody classes. However, this trend was not observed in the other compartments. We did not observe notable differences in the B cell activation markers CD69 and CD86 between the two strains, with the exception of mesenteric lymph nodes, where CD86 was decreased in the Bph/2 mice. However, within the mesenteric lymph node, there was a fair amount of variability in the Bpn/3 group and that trend was not noted in the other compartments. Overall, the data indicate decreased expression of IgD in splenic B cells from Bph/2 mice that may be due to increased antigen experience among the follicular B cells or differences in other B cell lineages.

Under homeostatic conditions, T cells can be defined as naïve or as one of several memory populations (11). While resident memory T cells are often found in tissues, such as the lung or gastrointestinal tract, effector memory and central memory T cells can be found in lymphoid organs (12, 30). All memory T cells have a lower threshold for activation compared to naïve T cells. While effector memory T cells gain effector function quickly after activation, central memory T cells take more time to become effector T cells. Conversely, central memory T cells are more proliferative than effector memory T cells in response to activation. Both effector and central memory T cells are more proliferative and induce greater levels of cytokine expression than naïve T cells. Within the CD4 population, our data demonstrate a decreased percentage of effector memory T cells, which was consistent across compartments, but most pronounced in spleen and mesenteric lymph nodes. Conversely, in the CD8 T cell population, there was a consistent decrease in the percentage of central memory T cells across all compartments. The decreased percentage of memory T cells in Bph/2 mice is consistent with, and could be largely responsible for, the decrease in T cell proliferation and cytokine induction in response to polyclonal activation.

There are some limitations in this study. Because this was our first investigation into Bph/2 mice, we kept the study somewhat limited in scope. For example, we are not able to determine sex differences because we only included male mice in this study. However, we intend to follow up with a subsequent study that will include both sexes. Our intention was to conduct a comprehensive assessment of baseline immunology in multiple lymphoid compartments. To assess immune cell composition, we used multiple flow cytometry panels, which allowed us to assess broad immune cell composition as well as B cell and T cell subpopulations. Using this approach, we found notable differences in T cell memory populations between the Bph/2 and Bpn/3 strains. However, this leads to the question of why this difference occurs. The development of memory can be impacted by numerous factors, including the strength of the primary response in naïve T cells, T cell exhaustion and expression of transcription factors that regulate memory, for example (31–33). It is also possible that memory T cells have migrated to another compartment in Bph/2 mice. None of these factors were addressed in the current study but would be interesting to pursue in future studies. Likewise, the differences in B cell antibody expression between the two strains could be due to differences in the threshold for B cell activation, which was not assessed in this study. Overall, the study pointed to a number of interesting immunological differences between the Bph/2 and Bpn/s mouse strains, but follow-up studies will be needed to understand why these differences occur.

A question that arises from the results of the present study is whether the Bph/2 model is a useful model for investigations into immune-driven hypertension. The Bph/2 model overall has many strengths, including that it is a stress-sensitive model, the hypertension occurs in both sexes (unlike many other models) and it is the only genetic model of hypertension in mice that is commercially available. There are some drawbacks to the Bph/2 model, however, including the fact that this model has not been completely characterized-- there are far fewer papers published in Bph/2 mice as compared to hypertension driven by angiotensin II infusion, for example. Importantly, there have been almost no published studies investigating differences in inflammation and immunity between hypertensive Bph/2 mice and the normotensive Bpn/3 mice (until our present study).

Our hypothesis in the current study was that immune cells from Bph/2 mice would be more responsive to polyclonal stimuli than those from Bpn/3 mice. Clearly, our data do not support this hypothesis but rather showed the opposite. While immune cell composition was similar between the strains, splenic immune cells from Bph/2 mice are hyporesponsive to T cell-specific stimuli as compared to those from Bpn/3 mice. Of interest, our data from Bph/2 mice resemble the early immune characterization of the spontaneously hypertensive rat (SHR) model. In particular, the initial characterizations of the immune system in SHR rats demonstrated that splenic immune cells were markedly hyporesponsive to polyclonal stimuli, which is consistent with our observations in the Bph/2 model (34, 35). In contrast, subsequent studies provided strong evidence for a role for the immune system in the development of hypertension in the SHR model. Specifically, treatment of SHR rats with immunosuppressive drugs, such as MMF and cyclophosphamide, mitigated the rise in blood pressure (36, 37). Of importance, increased expression of pro-inflammatory genes in the kidneys suggests that the contribution of the immune system to the development of hypertension in SHR rats is due to localized immune responses in the kidney and possibly other organs, such as brain and/or heart (38). Nonetheless, the early studies in splenic immune cells of SHR rats were important because in combination with later studies, they established that the hypertension occurs as a result of localized, rather than systemic, inflammation. Thus, while our present study is a comprehensive analysis of the secondary lymphoid organs in Bph/2 mice, it is only one step in a series of studies that will be needed to fully characterize the contribution of the immune system to the development of hypertension in this model.

The overall scientific question that this study aimed to address was whether Bph/2 and Bpn/3 mice share similar immune cell composition and similar T cell and B cell functionality. While immune cell composition is similar, the functionality of the lymphocytes differs between the two strains. There are notable functional differences in the lymphocyte populations. While the physiological significance of these differences is not yet clear, particularly with regard to the development of chronic diseases like hypertension, the data strongly suggest notable immune differences in these strains that could have long-term implications on immunity and inflammation.

## Data Availability

The original contributions presented in the study are included in the article/supplementary material. Further inquiries can be directed to the corresponding author.
